# Association of narcolepsy-cataplexy with *HLA-DRB1 and DQB1 *in Mexican patients: A relationship between HLA and gender is suggested

**DOI:** 10.1186/1471-2350-9-79

**Published:** 2008-08-15

**Authors:** Carmen Alaez, Ling Lin, Hilario Flores-A, Miriam Vazquez, Andrea Munguia, Emmanuel Mignot, Reyes Haro, Harry Baker, Clara Gorodezky

**Affiliations:** 1 Department of Immunology and Immunogenetics, InDRE, Secretary of Health; Mexico City, Mexico; 2 Center for Narcolepsy, Stanford University, Palo Alto, California, USA; 3 Instituto Nacional de Neurologia; Current Position: Sleep Disorders Clinics, UNAM; Mexico City, Mexico; 4 Sleep Disorders Clinic. Hospital Medica Sur; Mexico City, Mexico

## Abstract

**Background:**

Narcolepsy-cataplexy is characterized by excessive daytime sleepiness with recurrent episodes of irresistible sleep, cataplexy, hallucinations and sleep paralysis. Its aetiology is unknown, but it is positively associated with the human leukocyte antigens (HLA) in all studied populations. The purpose of the present study was to investigate the association of HLA class II *DRB1*/*DQB1 *alleles with narcolepsy-cataplexy in Mexican Mestizo patients.

**Methods:**

This is a case-control study of consecutive patients and ethnically matched controls. We included 32 patients diagnosed with typical narcolepsy-cataplexy, of the National Institute of Neurology, of the Institute of Psychiatry and at the Center of Narcolepsy at Stanford University. As healthy controls, 203 Mexican Mestizos were included. *DRB1 *alleles were identified using sequence based typing. A PCR-SSOP reverse dot blot was used for *DQB1 *typing. Allele frequency was calculated by direct counting and the significance of the differences was assessed using the Yates Chi square. Odds ratio and confidence intervals were evaluated.

**Results:**

HLA-DRB1*1501 (OR = 8.2; pc < 0.0001) and DQB1*0602 (OR = 8.4; pc < 0.0001) were found positively associated with narcolepsy. When deleting DQB1*0602+ patients from the analysis, DQB1*0301 was also found increased (OR = 2.7; p = 0.035; pc = NS). DQB1*0602/DQB1*0301 genotype was present in 15.6% of the cases (OR = 11.5; p = 0.00035), conferring a high risk. DRB1*0407 (OR = 0.2; p = 0.016 pc = NS) and DQB1*0302(OR = 0.4; p = 0.017, pc = NS) were found decreased in the patients. The gender stratification analysis showed a higher risk in females carrying DRB1*1501 (OR = 15.8, pc < 0.0001) and DQB1*0602 (OR = 19.8, pc < 0.0001) than in males (OR = 5.0 for both alleles; p = 0.012, pc = NS for *DRB1 *& p = 0.0012, pc = 0.017 for *DQB1*). The susceptibility alleles found in Mexicans with narcolepsy are also present in Japanese and Caucasians; DRB1*04 linked protection has also been shown in Koreans. A stronger HLA association is suggested in females, in accordance with the sexual dimorphism claimed previously.

**Conclusion:**

This knowledge may contribute to a better understanding of the disease pathogenesis in different populations. The evaluation of the risk to develop narcolepsy-cataplexy in carriers of the described alleles/genotypes may also be possible. A larger sample should be analysed in Mexican and in other Hispanic patients to confirm these results.

## Background

Narcolepsy is a serious chronic neurological sleep disorder affecting between 0.02–0.05% of the general Caucasian population [1]. It is characterized by excessive daytime sleepiness, cataplexy, hypnagogic hallucination, sleep paralysis, and nocturnal fragmented/disorganized sleep [2,3]. Narcolepsy causes cognitive dysfunction, low academic performance and interpersonal problems [4]. Cataplexy is a pathognomonic clinical symptom required for the diagnosis of narcolepsy, according to the International Classification of Sleep Disorders [5]. It is a symptom that occurs when the muscle tension, in various areas of the body, is suddenly decreased involuntarily and lasts from few seconds to several minutes. Cataplexy is generally induced by laughter, excitement anger, and other emotional changes [5]. Mutations in genes of the hypocretin (orexin) neurotransmitter system cause narcoleptic symptoms in animal models [6]. Although most patients with narcolepsy-cataplexy have a reduction of hypocretin concentration in the cerebrospinal fluid [7], mutations or polymorphisms in hypocretin-related genes are extremely rare [8]. A potential autoimmune mechanism has been suggested, supported by the finding that in most narcolepsy patients, 80–90% of hypocretin cells in the hypothalamus were destroyed [9]. The lost of hypocretin neurons [10] has also been shown in a few post-mortem cases. In addition, the transcription of preprohypocretin mRNA was significantly decreased in the brain of these patients [11]. This wake-promoting neuropeptide is involved in sleep regulation, energy homeostasis, reward-seeking, learning, and memory; and it is also involved in the body temperature regulation, endocrine function, and the cardiovascular system, among other systems [12]. Recent studies also indicate that hypocretin/orexin neurons can alter their intrinsic electrical activity according to fluctuations in the levels of nutrients and appetite-regulating hormones [13].

Narcolepsy transmission is polygenic, environmentally influenced and genetic factors play an important role in its expression. Family studies indicate the presence of a 20–40 times increased risk of disease expression in first-degree relatives, and monozygotic twin studies showed that concordance is partial (25–31%) [1]. Early onset French and Chinese patients suffering from narcolepsy have shown a positive family history as compared with late-onset patients [14,15], suggesting that the disease may be more likely due to genetic factors in this subgroup of patients.

Based on the strong HLA association, family study segregation [16,17] and the finding of reduction of hypocretin-1 levels in the cerebrospinal fluid of DQB1*0602 positive patients [18,19], an autoimmune mediated destruction of hypothalamic neurons secreting hypocretin has been suggested as the cause of the disease. However, serum autoantibody markers have not been detected and no immunological abnormalities have been found in patients with narcolepsy [20].

The disease is strongly associated in Caucasians and Japanese with the DRB1*1501-DQA1*0102-DQB1*0602 haplotype. In African Americans, it is associated with DQB1*0602 haplotypes bearing different *DRB1 *alleles (*1101 and *1503) suggesting that *DQA1 *and *DQB1 *play a primary role in susceptibility [17]. DQB1*0602 rather than DRB1*1501 has been found increased in the patients, indicating that the disease susceptibility allele or locus is within, or, in the vicinity of the DQ region [21]. Haplotype analysis and contiguous genomic sequencing across the region have identified no other candidate gene [22]. Two-five fold increased risk in DQB1*0602 homozygous vs. heterozygote patients has been demonstrated in different ethnic groups [23]. DQB1*0301/*0602 carriers are also at an increased risk, whereas DQB1*0602/*0601 and DQB1*0602/*0501 heterozygotes have a lower disease risk [23-25].

Several authors reported that 85 to 95% of patients with narcolepsy carry DQB1*0602 when cataplexy is clinically typical or severe. However, only 40–60% of the patients are DQB1*0602 when mild, atypical or no cataplexy exists [25,26]. TNF alpha region has also been claimed to be involved in susceptibility independently of class II loci [27].

Genetic factors in other chromosomes have also been implicated [28]. A gender dimorphism and a strong effect of the catechol-O-methyltransferase (*COMT*) genes seem to influence symptoms. *COMT *genotype distribution between male and female patients was associated with the response to modafinil in Caucasians, since the optimal dose of modafinil was approximately 100 mg lower in females with narcolepsy, suggesting that females are better responders to the drug [29].

The HLA genetic profile of Mexican and other Central American patients has not been published; therefore the aim of this study was to investigate the class II-*DRB1*/*DQB1 *allele distribution in a group of sporadic Mexican Mestizo patients with narcolepsy and to explore if the HLA association is gender related.

## Methods

### Subjects

A total of 32 patients (14 males (45.17%) and 17 females (54.83%) were included. (The gender of one patient was unknown). All the cases were chosen and diagnosed based on the International Classification of Sleep Disorders (ICSD) [5] using clinical histories, nocturnal polysomnography, and Multiple Sleep Latency Tests. It is important to emphasize that the inclusion and exclusion criteria were given to every participant in the Narcolepsy Component of the 13^th ^International Histocompatibility Workshop, and every centre must have used the same questionnaire and the same classification given by the experts [17]. The study was approved by the Ethics and Research Committees of each hospital. An informed consent was signed by every patient included in the study. Nine patients were selected from the Sleep Clinic at The Instituto Nacional de Neurologia in Mexico City, 12 were diagnosed at Stanford University Sleep Centre, and 11 were diagnosed at the Hospital Medica Sur in Mexico City. All patients were selected under exactly the same criteria in terms of ethnicity and diagnosis. Two hundred and three healthy controls belonging to the same population were included for comparison. These controls were all selected at the Department of Immunology and Immunogenetics in Mexico City and had no personal or family antecedents of narcolepsy. The patients and controls were all Mexican Mestizos defined according to the criteria of the Instituto de Investigaciones Antropologicas of the Universidad Nacional Autonoma de Mexico, UNAM [30]. All of them were Mexicans with at least parents, grandparents and great-grandparents born in Mexico; having Hispanic last names. Any individual with a non Hispanic background was excluded from the study. This ethnic group is the result of the admixture of Mediterranean, Black and Native genes [30,31]. The patients diagnosed in the USA followed exactly the same criteria, and most of them were in fact, patients from Mexico City from a previous collaborative done by Mignot E and Baker H. (Personal Communication). The controls were healthy subjects with no history of HLA confirmed associated diseases. Subjects with excessive daytime sleepiness, other sleep disorders, circadian or mental disorders, medication or substance abuse were carefully excluded. Each subject underwent a detailed clinical interview and completed a set of questionnaires according to The International Classification of Sleep Disorders [5].

### HLA-*DRB1 *&*DQB1 *typing

All patients and controls were typed at the Immunogenetics Department in Mexico City with the same technology and under the same technical conditions for each technique used. Ten mL peripheral EDTA blood were drawn from each patient coming from the Mexican Institutions. DNA was isolated using proteinase K digestion; purification was achieved with phenol chlorophorm and isopropanol was used for precipitation. DNA of patients diagnosed at Stanford University, were sent to our laboratory in Mexico City for HLA typing. *DQB1 *was typed on DNA samples from 32 patients, using a commercial kit based on Polymerase Chain Reaction and hybridization with membrane immobilized Sequence Specific Oligonucleotide Probes (PCR-SSOP reverse dot blot). *DRB1 *Sequence Based Typing (SBT) was performed in all patients using AlleleSEQR *DRB1 *kits, kindly donated by Atria Genetics. *DRB1 *and *DQB1 *alleles were typed in the control samples by PCR-SSOP using a chemiluminescent detection method designed for the 13th IHW (International Histocompatibility Workshop) [32].

*DRB1 *typing was performed in 27/32 samples because the amount of DNA was not enough. In one patient gender was unknown; therefore gender stratification analysis included only 31 patients.

Haplotype assignment was done on the basis of the very well known *DRB1-DQB1 *linkage disequilibrium data published by us in Mexican Mestizos [31]. It was assumed that no blanks were present; when a single HLA allele was found; in this case, the subject was considered homozygous. Rare haplotype associations were taken into account, only when the complementary haplotype was perfectly defined.

### Statistical Analysis

Allele frequency was calculated by direct counting. The Chi square test with Yates correction was used to assess the statistical difference of the HLA allele distribution between patients and controls. Bonferroni correction of the p value (pc) was done multiplying the p value by the number of comparisons made (equal to the number of tested alleles). This correction made more stringent the statistical significance of the results. Odds ratio (OR), was calculated when a significant association between the particular allele and the disease was found. Confidence interval (CI) (95%) was defined for every statistical deviation and is shown in each table. HLA related gender stratification was done using the SPSS 11 software.

### Analysis of DQB1*0602 negative patients

To assess whether HLA alleles, other than DQB1*0602 were associated with narcolepsy, the allelic frequency between patients and controls was compared, after taking out from the analysis the DQB1*0602 positive patients.

## Results

### HLA Association

Only two *DRB1 *alleles were found significantly deviated in the patients (Table 1). DRB1*1501 was significantly increased (OR = 8.2; CI = 3.9–17.6; p < 0.0001; pc < 0.0001). The frequency of DRB1*0407 was significantly decreased in them (OR = 0.2; CI = 0.02–1.2; p = 0.016), although the significance was lost after Bonferroni correction. DQB1*0602 which is in linkage disequilibrium with DRB1*1501 was associated with susceptibility (OR = 8.4; CI = 4.3–16.6; p < 0.001; pc < 0.001) and the frequency of DQB1*0302 was decreased in the patients (Table 2).

**Table 1:** HLA-*DRB1 *distribution in Mexican Mestizo patients with narcolepsy and in healthy controls.

**Locus**	**Total Patients**		**Total Controls**						
DRB1*	**N = 27†**	**AF%**	**N = 203**	**AF%**	**X**^2^**Y**	**OR**	**(CI)**	**p**	**pc**
0101	0	0	11	2.7	0.6				
0102	0	0	17	4.2	1.3				
0103	0	0	2	0.5	0.3				
1001	1	1.8	3	0.7	0				
1101	3	5.6	12	3	0.4				
1102	0	0	4	1	0				
1103	0	0	1	0.2	1.4				
1104	2	3.7	9	2.2	0				
1201	0	0	4	1	0				
1301	0	0	9	2.2	0.3				
1302	1	1.8	10	2.5	0				
1303	2	3.7	4	1	1				
1304	1	1.8	1	0.2	0.3				
1305	0	0	1	0.2	1.4				
1401	0	0	5	1.2	0				
1402	2	3.7	14	3.4	0.1				
1406	0	0	24	5.9	2.3				
**1501**	**15**	**27.8**	**18**	**4.4**	**35.6**	**8.2**	**(3.9–17.6)**	**<0.0001**	**<0.0001**
1502	2	3.7	5	1.2	0.6				
1503	2	3.7	2	0.5	2.6				
1504	1	1.8	0	0	1.4				
1601	0	0	4	1	0				
1602	1	1.8	21	5.2	0.5				
0301	3	5.6	18	4.4	0				
0302	0	0	3	0.7	0.1				
0401	0	0	1	0.2	1.4				
0402	0	0	7	1.7	0.1				
0403	3	5.6	7	1.7	1.7				
0404	3	5.6	17	4.2	0				
0405	0	0	12	3	0.7				
**0407**	**1**	**1.8**	**60**	**14.8**	**5.8**	**0.2**	**(0.02–1.2)**	**0.016**	**NS**
0410	0	0	2	0.5	0.3				
0411	2	3.7	12	3	0				
0701	4	7.4	27	6.7	0				
0801	0	0	6	1.5	0.1				
0802	3	5.6	51	12.6	1.6				
0804	1	1.8	0	0	1.4				
0806	1	1.8	0	0	1.4				
0901	0	0	2	0.5	0.3				

AF: allele frequency; X^2^Y: Chi^2 ^value with Yates correction; OR: Odds Ratio; CI: Confidence Interval; pc: Bonferroni correction (the p value was multiplied by the total number of alleles tested); NS: Not significant; **†**5/32 patients were not typed for *DRB1 *locus.

**Table 2:** HLA-*DQB1 *distribution in Mexican Mestizo patients with narcolepsy and in healthy controls.

**Locus**	**Total Patients**		**Total Controls**						
DQB1*	**N = 32**	**AF%**	**N = 203**	**AF%**	**X**^2^**Y**	**OR**	**(CI)**	**p**	**pc**
0201	8	12.5	43	10.6	0.1				
0301	16	25.0	100	24.6	0.0				
**0302**	**8**	**12.5**	**111**	**27.3**	**5.7**	**0.4**	**(0.2–0.9)**	**0.017**	**NS**
0303	2	3.1	5	1.2	0.4				
0402	5	7.8	60	14.8	1.7				
0501	2	3.1	35	8.6	1.6				
0502	0	0	5	1.2	0.1				
0503	0	0	5	1.2	0.1				
0601	0	0	4	1	0.0				
**0602**	**21**	**32.8**	**22**	**5.4**	**46.7**	**8.4**	**(4.3–16.6)**	**<0.0001**	**<0.0001**
0603	0	0	7	1.7	0.3				
0604	0	0	7	1.7	0.3				
0605	1	1.6	1	0.2	0.2				
0609	1	1.6	1	0.2	0.2				

AF: allele frequency; X^2^Y: Chi^2 ^value with Yates correction; OR: Odds Ratio; CI: Confidence Interval; pc: Bonferroni correction (the p value was multiplied by the total number of alleles tested); NS: Not significant.

Upon withdrawal from the analysis of DQB1*0602 positive patients and controls, DQB1*0301 was found associated with the disease (Table 3) since 9/11 remaining patients were carriers of this allele (OR = 2.7; CI = 1.1–6.5; p = 0.0035; pc = Non significant (NS). We calculated the *DQB1 *genotype distribution in order to know the risk conferred by different combinations (Table 4). Only two genotypes were associated with narcolepsy: the highest risk was conferred by the combination DQB1*0602/DQB1*0301 (OR = 11.5; CI = 2.6–50.7; p = 0.00035), present in 15.6% (5/32) of the patients. DQB1*0602/X (excluding *0301) was also found significantly increased with a lower risk (OR = 9.5; CI = 4.1–21.9; p < 0.0001). This combination was positive in 50% of patients (16/32). No DQB1*0602 homozygotes were found.

**Table 3:** HLA-*DQB1 *distribution in Mexican Mestizo DQB1*0602 negative patients and controls.

**Locus**	**Patients**		**Controls**						
DQB1*	Λ**N = 11**	**AF%**	ς**N = 181**	**AF%**	**X**^2^**Y**	**OR**	**(CI)**	**p**	**pc**
0201	4	18.2	38	10.5	0.6				
**0301**	11**	**50**	**97**	**26.8**	**4.4**	**2.7**	**(1.1–6.5)**	**0.035**	**NS**
0302	3	13.6	104	28.7	1.7				
0303	0	0	5	1.4	0.2				
0402	2	9.1	55	15.2	0.2				
0501	1	4.5	33	9.1	0.1				
0502	0	0	5	1.4	0.2				
0503	0	0	5	1.4	0.2				
0601	0	0	4	1.1	0.3				
0603	0	0	7	1.9	0				
0604	0	0	7	1.9	0				
0605	1	4.5	1	0.3	1.4				
0609	0	0	1	0.3	1.4				

AF: allele frequency; X^2^Y: Chi^2 ^value with Yates correction; OR: Odds Ratio; CI: Confidence Interval; pc: Bonferroni correction (the p value was multiplied by the total number of alleles tested); NS: Not significant; Λ: 11/32 total patients were DQB1*0602 negative; ς: 181/203 total controls were DQB1*0602 negative. ** this number includes two DQB1*0301 homozygotes.

**Table 4:** HLA-*DQB1 *genotypes distribution in Mexican Mestizo patients with narcolepsy and controls.

DQB1* Genotypes	**N = 32**	**GF%**	**N = 203**	**GF%**	**X**^2^**Y**	**OR(CI)**	**p**
0602/X(not *0301)	16	50.0	19	9.4	32.9	9.5(4.1–21.9)	<0.0001
0602/0301	5	15.6	3	1.5	12.8	11.5(2.6–50.7)	0.00035
0301/0301	2	6.3	15	7.4	0.01		
0301/X(no 0602 or 0301)	9	28.1	82	40.4	1.3		
X/X	2	6.3	99	48.8	18.7	0.1(0.0–0.4)	<0.0001

GF: genotype frequency; X^2^Y: Chi^2 ^value with Yates correction; OR: Odds Ratio; CI: Confidence Interval.

### Gender Stratification

Although DRB1*1501 and DQB1*0602 were increased in both, females and males, the risk was higher in females: DRB1*1501 (OR = 15.8 CI = 4.5–55.7, p < 0.0001, pc < 0.0001 in females) vs. (OR = 5 CI = 1.6–15.4; p = 0.012, pc = NS in males) and DQB1*0602, (OR = 19.8, CI = 5.9–66.9, p < 0.0001, pc < 0.0001 in females) vs. (OR = 5, CI = 1.9–13, p = 0.0012, pc = 0.017 in males). DQB1*0302 was found significantly decreased only in males (OR = 0.3, CI = 0.1–1, p = 0.043, pc = NS).

## Discussion

The results of this study are considered preliminary, due to the small sample size. However, it is important to mention that we are not "Hypothesis generating" but "hypothesis testing". As demonstrated by many statistician experts in the HLA field: "If an association is detected in the first case, it can be tested and confirmed in the latter without having to correct in multiple comparisons" [33]. Nevertheless, future studies in Hispanic admixed populations are needed to confirm the presence of other HLA allele associations. It is worth to mention, that *DRB1*/*DQB1 *association has been tested and repeatedly demonstrated by many authors in different ethnic groups [23-25,34]; therefore, the associations shown here, are real. In this study, we report on the genetic profile in a sample of narcoleptic Mestizo patients. DRB1*1501 (OR = 8.2; pc < 0.0001) and DQB1*0602 (OR = 8.4; pc < 0.0001) were the strongest associated alleles found in narcoleptic Mexicans, similarly to African, White Americans and different Oriental groups [23-25,34-37]. Fifteen of the 27 *DRB1 *typed patients, were positive for DRB1*1501 (allele frequency = 27.8 vs. 4.4% in the controls) and 21 of the 32 *DQB1 *typed patients were positive for DQB1*0602 (allele frequency = 32.8% vs. 5.4% in the controls). In five *DQB1 *typed patients, *DRB1 *was not typed because of insufficient DNA, thus we were not able to assemble the *DRB1*-*DQB1 *combinations in them. Interestingly, the DRB1*1501-DQB1*0602 haplotype was present in most of the DQB1*0602 positive patients but other DR2 allele combinations were also found. Indeed, two narcoleptic patients had DRB1*1502-DQB1*0602 and two had DRB1*1503-DQB1*0602. A DRB1*1503 but not DRB1*1502 association with DQB1*0602 has been reported in patients from Martinique [38] and in African Americans [23]. These results show, beyond doubt, that DQB1*0602, rather than DRB1*1501 is the major narcolepsy susceptibility allele in Mestizos. Interestingly, none of the DR52 associated-DQB1*0602 haplotypes were found in Mexican patients, but their frequency is low in the general healthy population. As an example, in 160 Mexican Mestizo healthy individuals typed in our laboratory, the haplotype frequency was 0.31% for each of the following combinations: DRB1*1101-DQB1*0602, DRB1*1201-DQB1*0602 and DRB1*1301-DQB1*0602 (unpublished data). Some of these haplotypes have been reported in narcoleptic patients in other populations, most notably in African Americans [23].

No increase in DQB1*0602 homozygosity was found, as previously reported in White Americans and in African American patients, in whom a two to four fold higher risk has been described, compared to heterozygotes [23]. The same has been shown in Japanese patients [24]. This fact cannot be explained only based on the different allele frequencies of DQB1*0602 across ethnic groups, since the frequency shown in Japanese (AF = 6.4%) [24] was similar to the one found in the control group of the present study (AF = 5.5%). The lack of homozygote patients may be due to the reduced number of cases. Thus, again, a larger number is needed to confirm these results.

As in other populations, other *DQB1 *alleles, beside DQB1*0602, influence narcolepsy susceptibility [24,39]. The analysis of *DQB1 *distribution in DQB1*0602 negative patients showed that DQB1*0301 (OR = 2.7, p = 0.03) was significantly increased in this subgroup of patients. DQB1*0301 had also the second strongest susceptibility effect, after DQB1*0602 in Africans, Japanese and White Americans [24]. In Mexican patients, DQB1*0301 occurred in the context of several HLA haplotypes that included DRB1*1101, *1303, *1304, *0806 and *1602; however the number of patients in this group was insufficient to perform additional comparisons and to explain the possible independent contribution of the mentioned *DRB1 *alleles to susceptibility. None of these patients had the DRB1*04-DQB1*0301, found associated in White Americans [24]. Genotype distribution in patients and controls showed that DQB1*0602/DQB1*0301 conferred the highest risk for susceptibility (OR = 11.4) compared to DQB1*0602/X (non *0301) (OR = 9.4). The former combination was also described as the one with the highest risk for the development of narcolepsy, across three different ethnic groups [24].

DRB1*0407, which is the most frequent allele in Mexican population, seemed to be linked to protection in the present study. This allele is in strong linkage disequilibrium with DQB1*0302 in Mexicans and the haplotype frequency of DRB1*0407-DQB1*0302 is 13.1% [31]. In Koreans DRB1*0406-DQB1*0302 was found protective in patients with narcolepsy [39]. None of these studies confirmed a possible effect of DRB1*04 in susceptibility as previously shown in Whites and Japanese [24]. It may be claimed that the *DQB1 *locus may also be involved in protection, since in Korean as well as in Mexican patients, DQB1*0302 was decreased, although combined with *0406 in Koreans and with *0407 in Mexicans. DQB1*0601 has also been associated with protection in Koreans [25,39] and Japanese [40], but the latter was not related with protection in Mexicans, perhaps due to its low frequency (AF = 0.5%) [31]. Similar findings have been published recently in Koreans, where again, DRB1*0406 was found negatively associated and DQB1*0301 was described as a susceptibility allele. The authors claim based on their own work, and previous work, that a remarkable consistency of the HLA association pattern across multiple ethnic groups and cultures exists [41]. Thus, even if our sample size is small and the results of protection and secondary association may be regarded as preliminary, our data are consistent with those published [21,23-26,39,41].

Interestingly, the overall positive rate for DQB1*0602 in Mexican patients was 65.6%, while in Japanese, White Americans and African Americans; the rate is between 75–80% [24]. To analyse if this difference was significant or not, we performed a statistical comparison between patients and controls from the present study and those from the Mignot et al. study [24]. No significant difference, regarding DQB1*0602 distribution, was found between Japanese and Mexican controls but we did find a significant deviation when comparing Mexican controls with White or African Americans healthy people (p = 0.001 and p = 0.00000001, respectively). These differences are due, undoubtedly, to the lower frequency of DQB1*0602 existing in the general Mexican population [42], which is similar to the one found in Orientals, but it is lower than the frequency in Caucasian and African Americans [43]. The lower frequency of the DQB1*0602 allele in Mexican patients compared with African (p = 0.0007), Caucasian (p = 0.003) and Japanese (p = 0.0009) patients, is due to our small number of cases.

Gender stratification showed a differential distribution of *DRB1 *and *DQB1 *alleles. The patients selection was unbiased since no significant difference was found when distribution of males Vs. females was compared (Z = 0.561, p = 0.29). To demonstrate if the gender selection among the controls was biased, we performed several analyses comparing the mean allele frequency in the overall control group with the frequency for HLA DQB1*0602 among male and female patients. However, the distribution of DQB1*0602 alleles among female and male controls was found in the limit of the significance (X^2^Y = 3.841, p = 0.05; data not shown). Therefore, it must be mentioned that even if the p value was p = 0.05, the control selection may have been biased. When HLA distribution was compared matching for gender, DRB1*1501 (OR = 15.8; pc < 0.0001) and DQB1*0602 (OR = 19.8; p < 0.0001) showed a higher risk in female patients, although the same alleles were significantly increased in males, but with less intensity. This stronger female HLA association was described for the first time. However, it is important to confirm these data with a larger sample size and in different ethnic groups. Thus, the gender association reported here should be regarded as preliminary. Even if we have a stronger HLA *DRB1*/*DQB1 *association in females, this does not imply, by any means, that females are more or less affected than males; this only means that there is a stronger HLA genetic predisposition to narcolepsy in females than in males. A better response in affected women to certain drugs [29] is not contradictory at all, with the HLA/female association.

The explanation for this gender specific association is not clear, but a sexual dimorphism and a strong effect of the COMT genotype on disease severity and response to modafinil have been shown [44]. Females with narcolepsy with high COMT activity fell asleep twice as fast as those with low COMT activity during the multiple sleep latency test, while the opposite was true for men [45]. A gender difference in body weight gain and leptin signaling in hypocretin/orexin deficient mouse models has been also claimed [46]. Obesity was more prominent in females in both preprohypocretin knockout mice and orexin/ataxin-3 transgenic narcoleptic mice and was associated with higher serum leptin levels, suggesting a partial leptin resistance [46].

## Conclusion

In conclusion, this is the first study reported in Mexican patients with narcolepsy-cataplexy; the profile of HLA class II allele associations was found to be complex. As in other diseases such as type I diabetes, multiple sclerosis and others, studied by our group in Mexican Mestizos, the HLA pattern in these diseases was somewhat distinct from the association typically found in Caucasians or Blacks and Orientals [47,48], illustrating the importance of analysing MHC associations in populations with different ethnic backgrounds. We also demonstrated an HLA associated sexual dimorphism in this population and a protective allele effect which was also shown in some Oriental populations. Insights on the different HLA associations in different ethnic groups may prove to be an important asset in the investigation of genetic factors and the molecular mechanisms of disease expression. This knowledge may be important for the design of predictive, therapeutic and perhaps preventive approaches.

## Competing interests

The authors declare that they have no competing interests.

## Authors' contributions

CA Drafted the manuscript and supervised the laboratory work. LL Collaborated in drafting the manuscript and extracted the DNA at Stanford University. HF-A Carry out the statistical analysis and revised the drafted manuscript. MV Collaborated with the technical HLA DNA typing and revised the drafted manuscript. AM Collaborated with the technical HLA DNA typing and revised the drafted manuscript. EM Selected the clinical cases and wrote the criteria for all Centres and for the 13th International Histocompatibility Workshop and revised the drafted manuscript. RH Diagnosed and selected the cases from The Instituto Nacional de Neurologia and revised the drafted manuscript. HB Diagnosed and selected the cases from and revised the drafted manuscript. CG Coordinated all the work, revised the results and statistical analysis, revised the drafted manuscript thoroughly; revised and corrected the final version. All authors read and approved the final manuscript.

## Pre-publication history

The pre-publication history for this paper can be accessed here:


